# Dyslipidemia and rupture risk of intracranial aneurysms—a systematic review

**DOI:** 10.1007/s10143-021-01515-3

**Published:** 2021-03-11

**Authors:** Katja Løvik, Johnny Laupsa-Borge, Nicola Logallo, Christian A. Helland

**Affiliations:** 1 Department of Clinical Medicine, University of Bergen, Bergen, Norway; 2 Department of Clinical Science, University of Bergen, Bergen, Norway; 3 Department of Neurosurgery, Haukeland University Hospital, Bergen, Norway

**Keywords:** Aneurysmal subarachnoid hemorrhage, Dyslipidemia, Hypercholesterolemia, Coronary artery disease, Intracranial aneurysms

## Abstract

Dyslipidemia is a well-established risk factor for coronary artery disease. However, the effect on cerebral artery disease, and more specifically the rupture risk of intracranial aneurysms, is unclear and has not yet been reviewed. We therefore performed a systematic review to investigate associations between different types of dyslipidemia and incidence of aneurysmal subarachnoid hemorrhage (aSAH). We used the MEDLINE, Embase, and Web of Science databases to identify clinical trials that compared the rupture risk among SAH patients with or without dyslipidemia. The risk of bias in each included study was evaluated using the Critical Appraisal Skills Program (CASP). Of 149 unique citations from the initial literature search, five clinical trials with a case-control design met our eligibility criteria. These studies compared aSAH patients to patients with unruptured aneurysms and found an overall inverse relationship between hypercholesterolemia and rupture risk of intracranial aneurysms. The quality assessment classified all included studies as high risk of bias. The evidence indicates that hypercholesterolemia is associated with a reduced rupture risk of intracranial aneurysms. However, it is not clear whether this relation is due to the dyslipidemic condition itself or the use of antihyperlipidemic medication.

## Introduction

The prevalence of intracranial aneurysms (IA) in the general population is estimated to be 1–2%, whereas the annual incidence of aneurysmal subarachnoid hemorrhage (aSAH) is estimated at around 1/10,000 in Norway [27, 41]. aSAH is a life-threatening condition with mortality rates as high as 35%, and a significant morbidity in survivors. For a long time, the research on SAH was mostly focused on treatment options and outcomes, but for the last 20 years, risk factors like age, sex, hypertension, aneurysm size, and smoking have been established [17]. However, the etiology of aSAH and more specifically the rupture risk of intracranial aneurysms are not fully understood.

Dyslipidemia is a well-established risk factor for developing coronary artery disease (CAD) [15]. The term dyslipidemia covers a broad spectrum of lipid abnormalities. Studies have shown that elevated levels of total cholesterol (TC) and low-density lipoprotein cholesterol (LDL-C) are associated with cardiovascular events [13]. Increased very-low-density lipoprotein (VLDL) remnants, reflected by elevated triglycerides (TGs), increased small, dense low-density lipoprotein (sdLDL) particles, and reduced high-density lipoprotein cholesterol (HDL-C) levels, are termed the atherogenic lipid triad and are more common than other pathological patterns [11].

Several risk factors for CAD, such as hypertension and smoking, have been associated with the development and risk of rupture of an intracranial aneurysm [4, 17]. Except for a family history of aSAH, these cardiovascular risk factors have the highest population-attributable risk associated with aSAH [36].

However, hypercholesterolemia was associated with a seemingly paradoxical 40% reduction in the risk of aSAH in a review of case-control studies [12]. Furthermore, a recent systematic review on obesity and aSAH indicated a reduced risk of aneurysm rupture with increasing BMI but concluded that this seemingly paradoxical relation remains to be studied in more detail [33]. The same group performed a systematic review on cholesterol as a risk factor for aSAH and concluded that elevated TC levels increased the risk of aSAH among men [26]. No other systematic reviews or meta-analyses have investigated the relationship between dyslipidemia and the risk of intracranial aneurysmal rupture causing aSAH.

In this review, we aimed to explore associations between different types of dyslipidemia (high levels of TC, high levels of LDL-C, low levels of HDL-C, and/or high levels of TGs) and the rupture risk of intracranial aneurysms, searching for studies comparing patients with unruptured intracranial aneurysms (UIA) to patients with aSAH.

Over the last 30 years, the use of statins and other lipid-lowering drugs in the treatment of dyslipidemia has increased rapidly, and investigating dyslipidemia separately without the impact of statins is challenging [37]. Therefore, we also considered the effect of statin use on the risk of aneurysmal rupture.

## Materials and methods

### Literature searches and study selection

The PRISMA guidelines 2020 [32] have been guiding this systematic review, and we used the PICO framework to formulate the study question and identify selection criteria of eligible studies:

- P (population/patients): A case group of adults with aSAH and a control group of adults with confirmed UIA
- I (intervention or exposure; treatment, diagnosis, or observation): Adults with aSAH and dyslipidemia
- C (comparison/control): Adults with aSAH without dyslipidemia
- O (outcome): Rupture risk reported as odds ratio (OR), hazard ratio (HR), or risk ratio (RR) when comparing the incidence of aSAH among patients with and without dyslipidemia

One investigator (KL) conducted systematic literature searches of the PubMed (https://pubmed.ncbi.nlm.nih.gov), Embase (http://www.embase.com), and Web of Science (https://clarivate.com/products/web-of-science) databases up to March 3, 2020.

The PICO process resulted in the following search strings:

- PubMed: (subarachnoid hemorrhage[MeSH Terms] OR intracranial aneurysms[MeSH Terms]) AND (dyslipidemias[MeSH Terms] OR hypercholesterolemia[MeSH Terms]).
- Embase: dyslipidemia AND (subarachnoid hemorrhage OR intracranial aneurysm)—limit searches to “full text,” “human,” “english.”
- Web of Science: (TS=((subarachnoid hemorrhage OR intracranial aneurysm) AND (dyslipidemias OR hypercholesterolemia))) AND LANGUAGE: (English).

Case reports, case series, letters, commentaries, book chapters, animal studies, and descriptive studies without calculated risk estimates (HR, OR, or RR) were excluded, as well as studies on children and articles written in languages other than English.

Titles and abstracts of all retrieved articles from the primary search were reviewed to identify eligible studies, and the full-text version of these was then read and assessed before the final inclusion according to the PICO criteria. In addition, manual searches of the references from selected original research and review articles were conducted.

### Data extraction and quality assessment

We chose the Critical Appraisal Skills Program (CASP) to evaluate the research methods of previous studies [5]. Using the checklists, we categorized the studies into low, unclear, or high risk of bias based on domains we considered being the most important to evaluate study biases and methodological shortcomings. These are the five domains we chose:

1. Sudden-death aSAH: When using hospital-based registers, a selection bias may occur if aSAH patients who die before hospital admittance are not included in the study. The risk factor status in these patients is as important as in those hospitalized, and substantial data is lost when excluding these patients. Inclusion of sudden-death aSAH was thus required to reach low risk of bias.
2. Sufficient sample size: The impact of dyslipidemia on rupture risk of intracranial aneurysms is not confirmed. To obtain statistically significant results, the number of patients included in a study matters. To calculate a sufficient sample size, we used a *Z*-score of 1.96 (corresponding to a 95% confidence interval [CI]), a standard deviation (SD) of 0.5, a margin of error of 5% and the following formula [8]:
  (*Z*-score)^2^ * SD * (1−SD) / (margin of error)^2^
  This resulted in a sample size of at least 385 patients.
3. Controlling for treatment of dyslipidemia: An unsolved question is whether an association between dyslipidemia and aneurysmal rupture risk is related to the condition of dyslipidemia per se or is due to treatment effects of lipid-lowering drugs. Therefore, to reach low risk of bias, the studies had to control for the use of statins and/or other medications in the statistical analyses, as far as possible, and to consider the contribution of treatment effects when discussing the relationship between dyslipidemia and rupture risk.
4. Confirmation of aneurysmal presence: The method of confirming that the intracranial hemorrhage was actually related to a ruptured aneurysm was chosen as a domain where possible bias could occur, as paroxysmal or traumatic SAH would provide wrong results when studying rupture risk. CT angiography, MRI with MR-angiography, or conventional angiography performed by an experienced radiologist was required to reach low risk of bias.
5. History of ruptured aneurysms: A previous incidence of aneurysmal rupture increases the risk of new ruptures. Therefore, patients with a history of any intracranial hemorrhage both in the UIA and aSAH group should be excluded to reach low risk of bias.

## Results

### Study selection

**Fig. 1:**
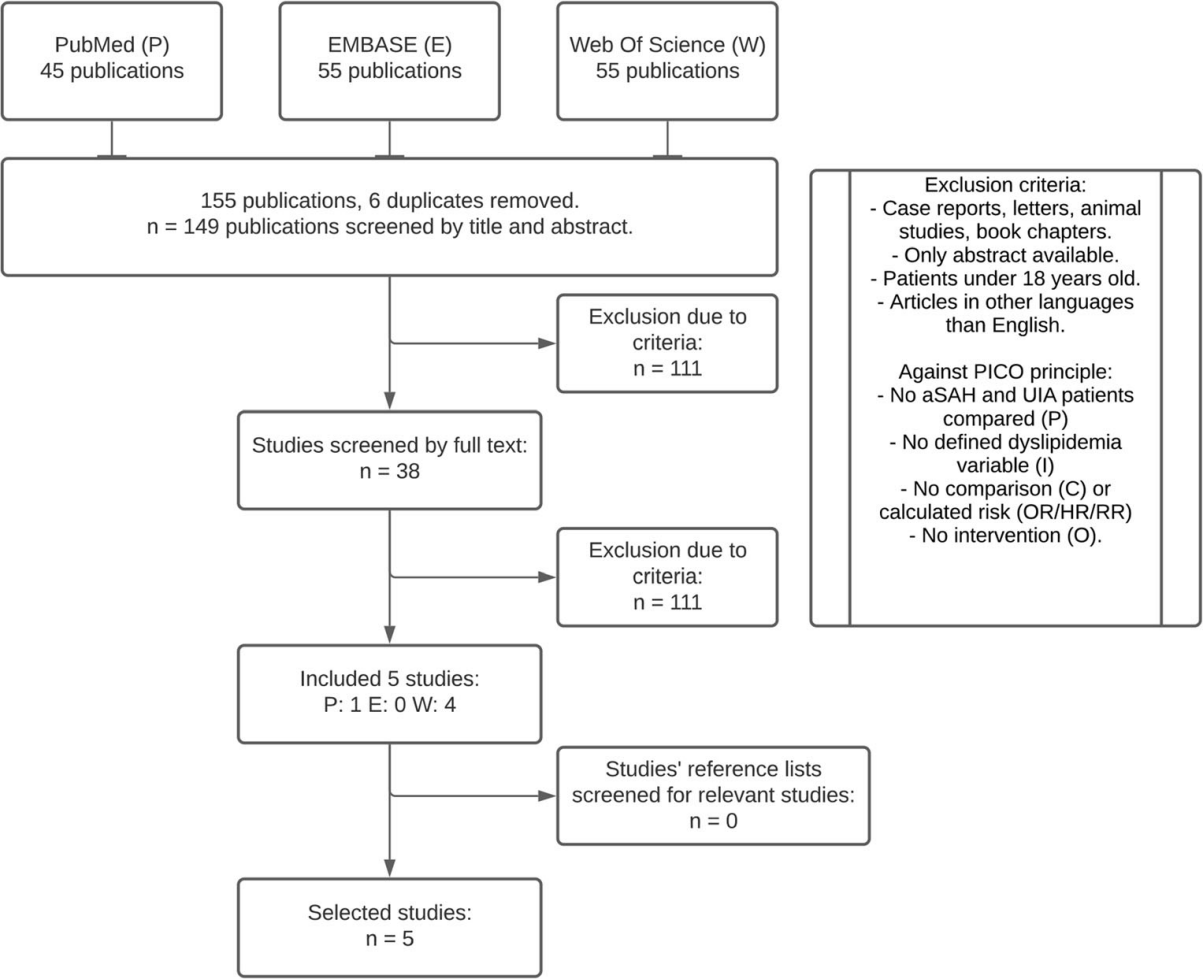
Flowchart of the literature searches and study selection. P, PubMed; E, Embase; W, Web of Science; aSAH, aneurysmal subarachnoid hemorrhage; UIA, unruptured intracranial aneurysms; OR, odds ratio; HR, hazard ratio; RR, relative risk. Made by K. Løvik in Lucidchart © 2021 Lucid Software Inc., based on the PRISMA flow diagram. Moher et al. [46]

A flowchart of the evaluated studies in this review is presented in Fig. 1. From 149 unique citations in the initial search, 111 articles were excluded after reviewing titles and abstracts. The remaining 38 studies considered relevant were then assessed in full text. Of these, five articles met the eligibility criteria and were included in the review [19, 22, 28, 42, 44].

Furthermore, we found six studies that were categorized as “near-misses,” i.e., considered relevant but did not meet all inclusion criteria [12, 21, 25, 29, 39, 40]. This was mainly due to the study design, meaning that they did not compare aSAH cases with UIA controls, but only investigated lipids as risk factors for aSAH, as in a previous systematic review [26]. From the reference lists of the five included studies, no other eligible articles were found.

### Study characteristics

All included studies had a case-control design [19, 22, 28, 42, 44], and compared aSAH patients to unmatched UIA controls in three different countries (Table 1).

**Table 1:** Study characteristics

First author	Country	Year	Study design	aSAH cases	UIA cases	Mean age aSAH	Mean age UIA	Sex (% male) aSAH	Sex (% male) UIA	Dyslipidemia definition
Yoshimura [44]	Japan	2014	Case-control	117	304	60.5	64,3	35	34.2	Statin use and hyperlipidemia in medical records
Vlak [42]	Netherlands	2013	Case-control	250	206	54.7	54.6	24.8	33	Diagnosis of hypercholesterolemia in medical records
Matsukawa [28]	Japan	2013	Case-control	78	62	58	63	34	31	TC >220 mg/dL and/or use of lipid-lowering medicaments
Inagawa [22]	Japan	2010	Case-control	858	798	64.0	65.9	34.6	34.6	TC >220 mg/dL and/or use of lipid-lowering medicaments
Hostettler [19]	UK	2018	Case-control	1729	605	53.2	57.0	29.7	29.8	Statin use and hypercholesterolemia in medical records

*aSAH*, aneurysmal subarachnoid hemorrhage; *UIA*, unruptured intracranial aneurysm; *TC*, total cholesterol

**Fig. 2:**
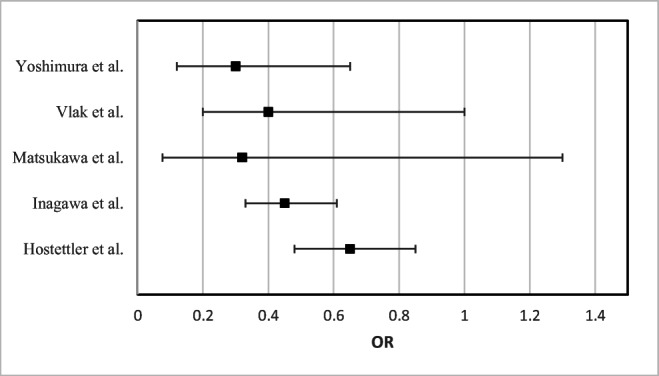
Forest plot of odds ratios (OR) from multivariable analyses. Yoshimura et al. adjusted for sex, age, hypertension, serum TC, smoking, and alcohol consumption. Vlak et al*.* adjusted for smoking, previous stroke, migraine, and a family history of aSAH. Matsukawa et al*.* adjusted for age, smoking, anterior and lateral dome direction of aneurysm, bleb, aneurysm size, and other unruptured aneurysm. Inagawa et al*.* adjusted for age and sex. Hostettler et al*.* adjusted for age, sex, ethnicity, smoking, aneurysm location, aneurysm size, and drug use including antihypertensiva, aspirin, and statin. Made by K. Løvik in Microsoft Excel, © 2021 Microsoft

One of these studies investigated only the rupture risk of a specific anatomic aneurysm location (anterior communicating artery) [28]. The results of the multivariable analyses from the five studies are presented in Fig 2.

#### Study 1

In a hospital-based case-control study in Japan, which investigated statin use as the primary exposure of interest, Yoshimura et al. showed that use of any statin was inversely associated with cerebral aneurysm rupture (OR 0.30, 95% CI 0.12–0.65) when controlling for sex, age, hypertension, serum TC, smoking, and alcohol consumption. [44]. The study population included 117 cases of patients with aSAH and 304 controls selected from patients with a newly diagnosed, unruptured aneurysm. In a stratified analysis by serum TC, the authors observed that statin use was inversely associated with cerebral aneurysm rupture regardless of serum cholesterol level in participants with 130–219 mg/dL (OR 0.21, 95% CI 0.08–0.55) and 220 mg/dL or more (OR 0.43, 95% CI 0.09–2.10), also when adjusting for sex, age, hypertension, smoking, and alcohol consumption.

#### Study 2

In a case-control study from the Netherlands, Vlak et al. found a decreased risk of rupture with hypercholesterolemia when studying 250 patients with aSAH compared to 206 patients with UIA in a univariable analysis (OR 0.3, 95% CI 0.1–0.5), as well as in a multivariable analysis including smoking, previous stroke, migraine, and a family history of aSAH (OR 0.4, 95% CI 0.2–1.0) [42]. Data from the questionnaire were insufficient to conclude whether hypercholesterolemia or its treatment with statins exerted a risk-reducing effect.

#### Study 3

Matsukawa et al. studied specifically the rupture of anterior communicating artery (ACOM) aneurysms in a case-control study including 78 patients with aSAH and 62 patients with UIA [28]. Hypercholesterolemia was defined as taking antihyperlipidemic agents or having a TC level of >220 mg/dL (>5.7 mmol/L). The study found that hypercholesterolemia was significantly more common in the control group with UIA compared to the aSAH patients using the Student t-test (*P* = 0.0092), but this relationship was not significant in a multivariable logistic regression (OR 0.32, 95% CI 0.076–1.3) with covariates including age < 60 years, current smoking, anterior and lateral dome direction, bleb, aneurysm size >5 mm, and another unruptured aneurysm.

#### Study 4

Another Japanese study conducted by Inagawa et al. in Shimane City included 858 patients with ruptured aneurysms, 285 patients with UIA (and no history of aSAH), and 798 healthy control subjects [22]. They found that hypercholesterolemia was strongly associated with a decreased risk of rupture, regardless of age and sex (OR 0.45, 95% CI 0.33–0.61). Hypercholesterolemia was also significantly more common in patients with UIA than in controls. aSAH was confirmed by CT scan, surgery, and/or autopsy. Hypercholesterolemia was considered present when the serum TC level was 220 mg/dL (5.7 mmol/L) or higher, and/or if patients were being treated with lipid-lowering medications such as statins. They did not report results from models controlling for drug use. Therefore, the impact of statins is unknown.

#### Study 5

Hostettler et al. compared patients from 22 UK hospitals, including 605 patients with UIA and 1729 patients with aSAH over a 3-year period [19]. Hypercholesterolemia was found to be inversely associated with aSAH in the univariable analysis (OR 0.45, 95% CI 0.37–0.55, *P* < 0.001), and in the multivariable analysis including age, sex, ethnicity, smoking, antihypertensive medication, aspirin use, aneurysm location, and aneurysm size as covariates, and independently of statin use (OR 0.64, 95% CI 0.48–0.85, *P* = 0.002).

#### Risk of bias and quality assessments

None of the studies comparing UIA to aSAH [19, 22, 28, 42, 44] fulfilled the criteria for low risk of bias classification in all of the chosen domains (Table 2). Thus, all five studies were overall classified as high risk of bias.

**Table 2:** Risk of bias

First author	Sudden-death aSAH	Sufficient sample size	Classification of dyslipidemia	Confirmation of aneurysmal presence	History of aSAH—exclusion	Risk of bias
Yoshimura [44]	−	+	+	+	+	High
Vlak [42]	−	+	−	+	+	High
Matsukawa [28]	−	−	−	+	+	High
Inagawa [22]	NA	+	−	+	+	High
Hostettler [19]	−	+	+	+	+	High

+ low risk of bias;− high risk of bias; *NA* (not applicable), unclear risk of bias; *aSAH*, aneurysmal subarachnoid hemorrhage

## Discussion

The five studies which compared UIA patients to aSAH patients found a statistically significant paradoxical relation between hypercholesterolemia and risk of aneurysmal rupture. Although one study was able to adjust for statin use, it is not clear whether the protective effect derives from the hypercholesterolemia itself, or the use of statin therapy.

A protective effect of hypercholesterolemia could partly emerge through stabilization of the aneurysm wall by formation of hard atherosclerotic plaque, and further preventing a newly formed aneurysm from rupture [19].

The traditional “diet heart” or lipid hypothesis consists of three main statements: (1) higher intakes of saturated fatty acids (SFAs) raise circulating levels of TC and LDL-C, (2) elevated blood levels of cholesterol lead to atherosclerosis and cardiovascular disease (CVD), and consequently (3) a diet rich in SFAs promotes atherosclerosis and increases the risk of CVD. This hypothesis has in the last 20 years been criticized and challenged by contradictory studies showing that cholesterol-lowering did not prevent CVD and that reduced intakes of dietary SFAs have not been consistently associated with reduced CVD events or mortality [2, 6, 7, 9, 18, 24, 30, 34, 45].

The most common cholesterol-lowering drugs are statins, and the number of patients on statin therapy is increasing. Statins reduce atherogenic lipoproteins, reduce C-reactive protein levels, and thereby have an anti-inflammatory effect which may be beneficial for the stabilization of the aneurysm wall [16]. It is also important to consider the side effects of statins in multiple organ systems, such as muscular problems, mental and neurological symptoms, liver damage, and renal failure [35]. Recent literature has suggested statin therapy in the progression of abdominal aortic aneurysmal disease, after animal studies showing a reduction in aortic diameter and the incidence of aneurysms [3, 38]. A future possible recommendation of statin prophylaxis in patients with high risk of aSAH should therefore be discussed with caution.

### Limitations and strengths in the reviewed studies

The main limitation in the reviewed studies, which is also relevant for the possible clinical relevance of the results, is the effect of statin use in the patients studied. If the use of statins exerts the apparent protective effect of dyslipidemia, this demands a different approach.

Considering the origin of the studies, three of them derived from Japan, which may reflect ethnic differences in the patient population given that recent genetic analyses have suggested that Japanese (and/or Finnish) patients are at higher risk for aneurysm rupture [20].

The authors of all five studies discussed the possibility of a selection bias, especially in the UIA group, due to the fact that a family history of aSAH is more likely to undergo brain imaging.

Considering sample sizes, two of the studies included large patient groups [19, 22], and one study had a sample size too small to be considered as low risk of bias [28]. Two studies have patient numbers within the low risk of bias classification, but still relatively small. The inclusion phase was, respectively, 5 years (2 years for the aSAH group) in one study [44] and 3 years in another study [42]. This may be explained by the decreasing incidence of aSAH worldwide, partially due to less smoking and lower blood pressure [10].

In the study by Yoshimura et al*.* (study 1), the primary exposure of interest was statin use [44]. They found that high cholesterol levels were protective against cerebral aneurysm rupture and that statins appeared to be protective against cerebral aneurysm rupture in high and normal cholesterol-level groups. An important limitation in this study was that they did not analyze serum lipids before and after statin administration to verify the effects of statins, but, regardless of serum cholesterol levels, statin use was inversely associated with the occurrence of aneurysmal rupture. The authors discussed that it remains uncertain whether statins are beneficial for the prevention of aSAH and requested further and larger studies to confirm their findings and safety of statins in aSAH prevention. The number of patients was within our classification of low risk of bias.

Vlak et al*.* (study 2) did not register the use of statins [42]. Whether the observed reduction of risk of rupture was caused by the use of statins or by the hypercholesterolemia itself is therefore unknown. The authors suggested that if the risk decreasing effect of hypercholesterolemia was mediated by statin use, statins might be an additional treatment option for decreasing the rupture risk of UIA. They also discussed the possible difficulties of performing a prospective study on the influence of drugs on the rupture risk, which would need a very long-term follow-up.

Matsukawa et al*.* (study 3) and Inagawa et al*.* (study 4) both defined hypercholesterolemia as taking antihyperlipidemic agents and/or having a TC level of >220 mg/dL [22, 28]. They were not able to correct for statin use, and the origin of the protective effect is unknown.

The study by Matsukawa and co-workers was conducted retrospectively at a single institution, with a patient number too small to draw definite conclusions [28]. Furthermore, they only studied anterior communicating artery (ACOM) aneurysms. This location has a significantly higher rupture rate than other anterior circulation aneurysms, where common anatomical variations such as unilateral A1 hypoplasia (or aplasia) further predisposes ACOM aneurysms to rupture. Thus, the results from this study may not be representative of aSAH risk in general [1]. Although the study found a reduced risk of rupture among patients with hypercholesterolemia, the authors did not suggest clinical implications or further studies on the topic, probably because the study was mainly focused on aneurysm characteristics.

Inagawa et al*.* proposed that hypercholesterolemia may contribute to aneurysm formation, whereas it may reduce the risk of aneurysm rupture [22]. Thus, they highlighted the important point that the risk factors for aneurysm formation and rupture may  not necessarily be the same.

The British study from Hostettler et al*.* (study 5) recorded both statin use and a diagnosis of hyperlipidemia [19]. They found hypercholesterolemia to be inversely associated with risk of aneurysmal rupture, independent of statin treatment. The authors suggested that part of the effect could emerge through stabilization of the aneurysm wall, preventing a newly formed aneurysm from rupturing. The main focus of the conclusion was the effect of aspirin use on prevention of aSAH, and the possible clinical implications regarding statins were not further discussed.

### Confounders in the reviewed studies

The main confounding factor in the reviewed studies is the use of statins and whether the apparent protective effect derives from this or the actual dyslipidemia.

The anti-inflammatory and antioxidant effect of statins may cause a profitable effect in patients with intracranial aneurysms. Lipid accumulation is known to occur in the wall of cerebral aneurysms, and this mechanism has been proposed as a component in aneurysm wall pathogenesis as it is in the pathophysiology of an atherosclerotic plaque [14, 23, 31].

The five reviewed studies have, to our knowledge, considered the most relevant risk factors in their statistical analyses. It is still possible that other less established or unknown factors may have affected the results, such as anthropometric variables like body mass index (BMI) and body fat percent, the concentrations of different lipoprotein particle subclasses, and apolipoproteins, but the study designs have not allowed a further investigation thereof.

## Conclusions

We were able to identify five studies investigating the association of dyslipidemia and the risk of aneurysmal rupture, which all described an inverse association when comparing aSAH patients to a control group with UIA. Whether this effect is due to dyslipidemia or medication for this condition is not fully understood, but one study found a protective effect independent of antihyperlipidemic agents [19].

Our assessment on risk of bias defined all five studies as low quality. Inclusion of sudden-death aSAH patients is a practical challenge and perhaps unrealistic to expect. Without this criterion, two studies would qualify as low risk of bias. Since all five studies describe a possible protective effect of dyslipidemia on aneurysmal rupture and discuss their results with caution, we consider the results relevant for future research and clinical treatment of UIA. We believe this review raises new important questions to be answered in further studies. Ideally, it would be interesting to study patients with dyslipidemia without use of statins, but this is practically and ethically difficult due to the globally increasing use of statin therapy [43]. Another suggestion is to perform a prospective study treating patients with established intracranial aneurysms with statin therapy and follow the risk of rupture.

## Data Availability

Not applicable.
